# Supplementary material for: Vibration, a treatment for migraine, linked to calpain driven changes in actin cytoskeleton
Source: PLoS One. 2022 Apr 28;17(4):e0262058. doi: 10.1371/journal.pone.0262058 (PMC9049534; doi:10.1371/journal.pone.0262058)
Supplement: S1 Raw images — (PDF) [file pone.0262058.s001.pdf]

Original gel for Figure 3

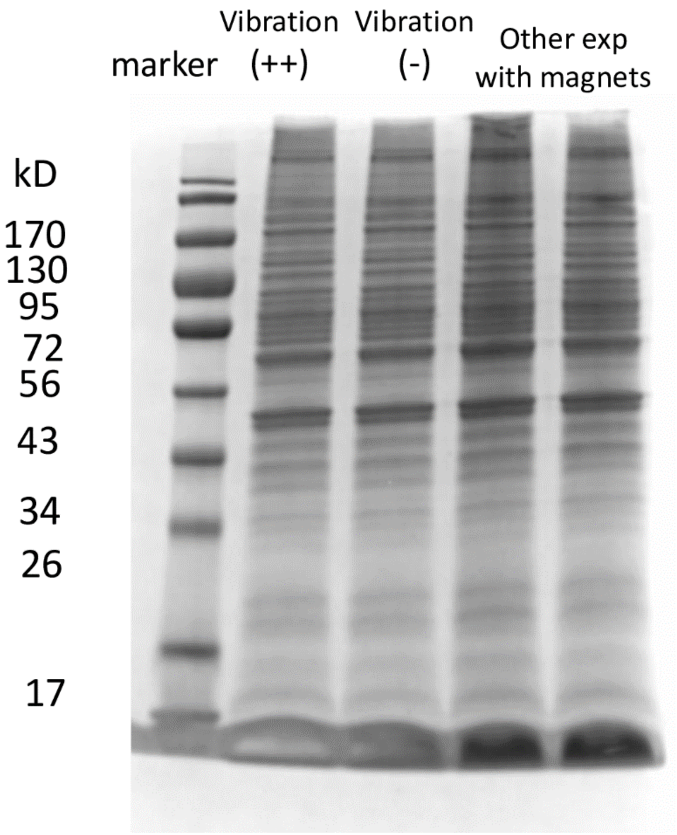

S3.2 Picture of Original Total Protein Gel from Experiment #2 [Mar 25]. Total protein bands of cytoplasmic lysates from HeLa cells with vibration [vibration (++) representing 1200 rpm] or without vibration (vibration -). Cells were lysed for total protein, separated by vertical electrophoresis and stained for total protein. Marker indicates 10 molecular marker bands corresponding to protein size. Bands from each lane were aligned based on relative front in comparison to relative front of marker bands. An arbitrary volume unit was assigned to each band by analysis software. Lanes 4 and 5 are samples from another experiment investigating magnetic force.

Original gel for Figure 4

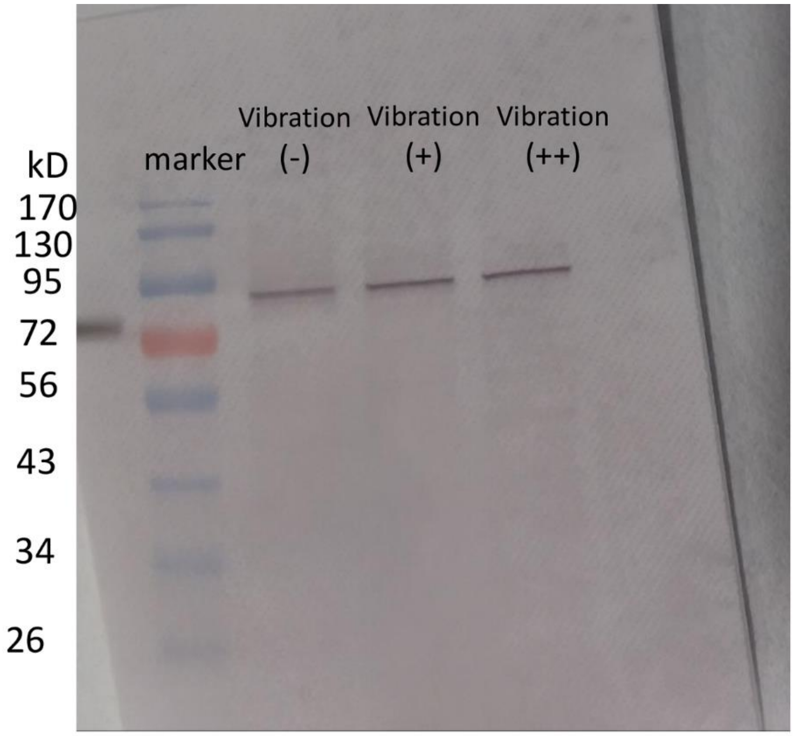

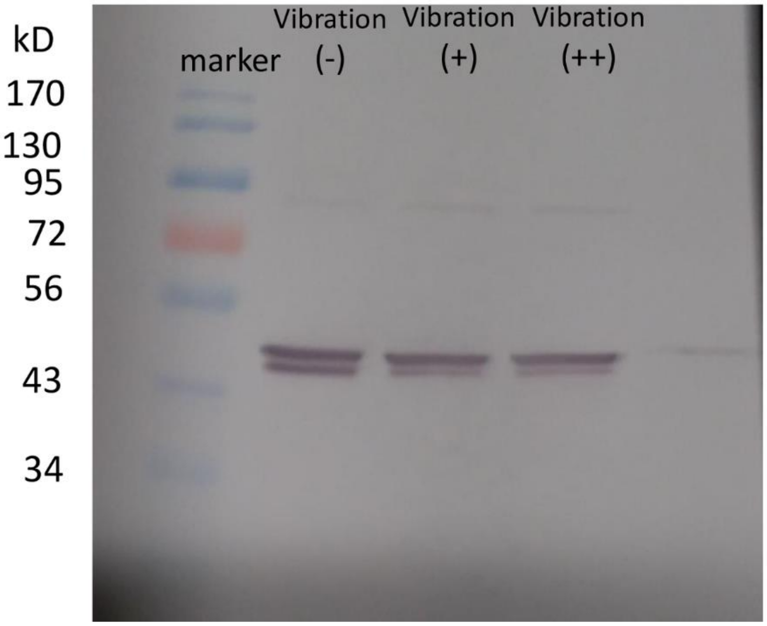

S4.3 Picture of Original Immunoblots from Experiment #3 [Dec 13]. Immunoblot of cytoplasmic lysates from HeLa cells directly from incubator (control), without vibration (vibration -) or with increasing levels of vibration [vibration (+) and vibration (++) representing 600 and 1200 rpm, respectively]. Lysates probed for calpain-1 [top panel] and beta-actin [bottom panel] as a loading control. Marker indicates molecular marker bands corresponding to protein size (kD).
