# Supplementary material for: Vibration, a treatment for migraine, linked to calpain driven changes in actin cytoskeleton
Source: PLoS One. 2022 Apr 28;17(4):e0262058. doi: 10.1371/journal.pone.0262058 (PMC9049534; doi:10.1371/journal.pone.0262058)
Supplement: Attachment — Submitted filename: Response to Reviewers minor.pdf [file pone.0262058.s003.pdf]

GRAND VIEW UNIVERSITY
College of Social and Natural Sciences
Department of Biology
Des Moines, IA 50316

March 31, 2022

RE: Response to Reviewers concerning PONE-D-21-39301

Dear Dr. Talamas-Rohana:

We greatly appreciate the opportunity to revise our manuscript PONE-D-21-39301 entitled "Vibration, a treatment for migraine, linked to calpain driven changes in actin cytoskeleton".

Journal request has been addressed. We have reviewed our reference list to ensure that it is complete and correct. We have not retracted any cited papers. However, we found two typographical errors that we have fixed (in reference number 42 the journal title listed twice and in reference number 44 the authors names were all capitals).

Response to Reviewer's Comments

Overall, we greatly appreciate the comments made by both reviewers that the manuscript has been improved to support the conclusion by including statistical analyses of the results. We have responded to both reviewer's recommendations as follows.

Reviewer #1

The comments made by reviewer #1 concerning the statistical analysis of data presented in figure 1 and figure 2 has been addressed. In this regard, we used a T-test, instead of an ANOVA, to analyze data presented in figures 1 and 2. As the result was the same, there were no changes made to the figures. However, we do want the reader to be clear as to what statistical analysis was performed.

Therefore, to clarify the statistical analysis, we added a comment ("Two-tailed T-test (Two-sample assuming equal variances) for analysis of cell area and filopodial length, which had only two groups") to the materials and methods. In addition, we updated the datasets with the new analysis at our public data repository. The new analyses (documents S1.4 Dataset Cell Shape Area Ttest and S2.5 Dataset Cell Actin Filament Ttest) can be found at the open science framework at the following link. https://osf.io/t64jg/?view_only=7f2c9fbd69004b4782aea68aa86fe736

Reviewer #2

In response to reviewer #2, we fixed all the typographical errors mentioned. We also found and fixed two additional spacing errors.

Sincerely,

A J LaGier

Adriana J. LaGier, Ph.D.
Associate Professor of Biology
