# Supplementary material for: Vibration, a treatment for migraine, linked to calpain driven changes in actin cytoskeleton
Source: PLoS One. 2022 Apr 28;17(4):e0262058. doi: 10.1371/journal.pone.0262058 (PMC9049534; doi:10.1371/journal.pone.0262058)
Supplement: Attachment — Submitted filename: Response to Reviewers.docx [file pone.0262058.s002.docx]

College of Social and Natural Sciences

Department of Biology

Des Moines, IA 50316

February 17, 2022

RE: Response to Reviewers concerning PONE-D-21-39301

Dear Dr. Talamas-Rohana:

We greatly appreciate the opportunity to revise our manuscript PONE-D-21-39301 entitled “Vibration, a treatment for migraine, linked to calpain driven changes in actin cytoskeleton”.

**All journal requirements have been addressed.**

1) We have ensured that the manuscript has met style requirements based on provided links, particularly appropriately formatted heading and figure labels and removing funding information from acknowledgements. 2) We have matched and provided grant numbers for the award received from Carver. 3) We have removed the four mentioned ‘data not shown’ references from the manuscript as the comments did not enhance the progression of ideas being presented by the study. 4) We have included a full ethics statement in the “Methods” section of the manuscript. 5) We have provided all original, uncropped and unadjusted images mentioned in the manuscript in a public data repository that can be found at the open science framework (OSF) at the following link. <https://osf.io/t64jg/?view_only=7f2c9fbd69004b4782aea68aa86fe736>

**Response to Reviewer’s Comments**

Based on the editor’s comments, we have responded to both reviewer’s recommendations as follows.

Reviewer #1

Overall, we greatly appreciate the comments made by reviewer #1 as they have enhanced the statistical soundness and data availability of our study. As an aside, this is our first experience publishing in an open access journal and we were previously unaware of public data repositories. In this regard, we have created an open access link to our data at OSF so that all data is electronically available.

Specifically, all comments made by reviewer #1 have been addressed.

1.- In regards to the data concerning vibrational impact on actin filament assembly, particularly filopodia (figure 2), we have embraced the reviewer’s comment that these structures are easily recognizable and quantifiable. Therefore, we replaced the staining intensity data with data where we measured the length of the filipodia (as suggested). As such, we replaced figure 2C with the updated figure.

2.- In regards to the data concerning vibration altering intracellular levels of protein (figure 3), we have added figure 3A, which shows the protein gel pattern in addition to the densitometry provided in the original version.

3.- In regards to the data concerning vibration increasing intracellular level of calpain protein (figure 4), we have added figure 4B, which is a densitometry analysis for three technical replicates. We are uncertain why the image had no captions as the figure submitted had these captions. However, perhaps now that the figure has been revised to include figure 4B, these captions are evident. In addition, reference to captions are within the figure legend. Additionally, all original, uncropped and unadjusted pictures of gels with annotations are provided in the public repository.

4.- We have fixed our oversight in regards to being explicit about experimental replication. The materials and methods and figure legends now reflect how many technical replicates were performed. Additionally, data from each replicate is provided in the public repository.

5.- In regards to the statistical analysis performed on the rounded phenotype data presented in figures 1 and 5, we have taken the reviewers suggestion and enhanced the statistical analysis of this data. For the revision, we have used an Excel data analysis package with XReal stats, to perform a one-factor ANOVA supplemented with a Tukey HSD post-hoc analysis within and between groups. The method for this analysis with the addition of technical replicates was updated in the materials and methods. As an aside, based on reviewer #2 comments, the ‘rounded phenotype’ counts were replaced with cell area.

We have revised the manuscript to include all of reviewer #1 comments and appreciate the comments which we believe have enhanced the statistical robustness and data availability of the manuscript.

Reviewer #2

Overall, we greatly appreciate reviewer #2’s comments that this study is of interest and could make a worthy contribution to understanding vibrational treatments. The comments concerning how to improve our subjective observations has substantially improved the quality of this work and we value the reviewer’s insight.

Specifically, all comments made by reviewer #2 have been addressed.

1) In regards to our observation that cells exposed to vibration were more susceptible to losing prominent filopodia (Figure 1), we initially assessed this phenomenon using a rounded phenotype. However, reviewer #2’s insight that cell area would be more quantifiable and less subjective was incredibly valuable. As such, we reassessed the data using cell area and have replaced the original figure 1C showing rounded phenotype with cell area data (revised Figure 1C). In addition, we have added a section “cell phenotype” to the materials and methods to clearly provide the criteria used to assess the ‘round phenotype’. We believe that this has provided more accurate and measurable results.

2) In regards to the data concerning vibrational impact on actin filament assembly, particularly filopodia (figure 2), we noted the reviewer’s comment that the fluorescence intensity per cell area would depend on image acquisition and implies an increase in the number of actin filaments. As we did not suppose that there was an increase of actin filaments, but rather a rearrangement of actin filaments corresponding with a retraction of filopodia, we understand the reviewer’s concerns. As such and based on a suggestion made by reviewer #1 that filopodia were easily recognizable, we have reassessed our data set by measuring filopodial length rather than fluorescence intensity. Therefore, we replaced the staining intensity data with data where we measured the length of the filipodia (replaced figure 2C with the updated figure). In addition, we added a section to the materials and methods to describe this process. We believe this has resolved the technical errors that may have accompanied our original assessment of the data. In addition, the statistical significance of the data was enhanced.

3) In regards to the statistical robustness of the data concerning vibration altering intracellular levels of protein (figure 3), we have added a representative picture of one of three gels (Figure 3A) and provided the average and error for three independent experiments (revised Figure 3 to figure 3B including error bars). The 2-D presentation of the graph was of concern because it impeded interpretation of the data. We realized that interpretation of this data would make it necessary to assess the vibration (+) band volumes in relation to the vibration (-) ones. Therefore, we graphed the data in one dimension utilizing the relative difference in volumes between vibration (+) and vibration (-). In addition, so that the gel picture and graph aligned, we converted the 2-D column graph to a 1-D bar graph. We agree with reviewer #2 that the single plane in addition to the incorporation of error bars facilitates the interpretation of data presented about intracellular levels of protein.

4) In regards to the data concerning vibration increasing intracellular level of calpain protein (figure 4), we have added figure 4B, which is a densitometric analysis of calpain levels in relation to loading controls and includes data from three independent experiments. The corresponding statistical analysis was provided in the figure legend and marked on the figure. Additionally, all original, uncropped and unadjusted pictures of the gels with annotations are provided in the public repository.

5) As in Figure 1, we have reassessed data concerning the ‘rounded phenotype’ in the absence or presence of calpain inhibitor (figure 5) utilizing the cell area criteria defined in this revision of the manuscript. The cell area criteria suggested by reviewer #2 is indeed more robust quantitatively and less subjective, as noted by the attenuated discrepancies between figure 1 and figure 5 data. As noted by the reviewer data from the original submission using the ‘rounded phenotype’ had vibration (-) cells at 7.5% rounded in figure 1 and 40% rounded in figure 5. Utilizing the cell area criteria vibration (-) cells had a 20.3 ± 1.6 area in figure 1 and 31.5 ± 12.1 area in figure 5. Additionally, data from the original submission using the ‘rounded phenotype’ had vibration (+) cells at 50% rounded in figure 1 and 75% rounded in figure 5. Utilizing the cell area criteria vibration (+) cells had a 10.8 ± 1.8 area in figure 1 and 9.1 ± 3.1 area in figure 5. As suggested by reviewer #2, we believe that the discrepancies noted in the original submission were a result of the use of a subjective criterion and that measuring cell area was useful to address these discrepancies. As such, Figure 1C and figure 5 from the original submission was replaced with Figure 1C and figure 5 in the revision which showed data using cell area.

6) In regards to data concerning vibration altering calpain gene expression, reviewer #2’s suggestion to analyze protein levels (figure 4) allowed us to compare how the increase of calpain transcript levels corresponded to an increase in protein levels. We determine that the change in protein levels was 2.7 ± 1.3 aligned well with the 4.8 ± 0.7 fold increase noted in calpain transcripts. We have added a comment to the discussion about this point.

In summation, we found great value in the reviewer’s comments. We believe that their expertise and insight has allowed us to revise the manuscript in ways that have transformed the study from being descriptive to being highly quantitative with objective criteria and robust statistical significance.

We greatly appreciate the reviewers comments that our work is relevant and an interesting topic of study because we are earnest in our belief that the study is of import.

Sincerely,

Adriana J. LaGier, Ph.D.

Associate Professor of Biology

alagier@grandview.edu

515-263-2874
